# Ultrasound characteristics and risk factors of female patients with pelvic varicose veins and concomitant chronic pelvic pain

**DOI:** 10.1016/j.jvsv.2024.102165

**Published:** 2024-12-27

**Authors:** Binyu Zheng, Gaorui Liu, Yong Liu

**Affiliations:** a Department of Ultrasound, Beijing Shijitan Hospital, Capital Medical University, Beijing, China; b Western Sydney Vascular, Westmead, New South Wales, Australia

**Keywords:** Chronic pelvic pain, Pelvic venous reflux, Ultrasound, Risk factor

## Abstract

**Objective:**

The study aims to elucidate clinical and ultrasonographic characteristics of female patients diagnosed with pelvic varicose veins (PVVs) and to assess potential risk factors associated with incidences of chronic pelvic pain (CPP) in this population.

**Methods:**

Clinical and ultrasound data were retrospectively collected from female patients with PVVs at Beijing Shijitan Hospital between December 2017 and October 2022. Patient cohorts were divided into two groups based on whether they had been experiencing non-periodic pelvic pain over 6 months, consistent with the symptoms of CPP. Comparative analyses were conducted between the two groups, utilizing both univariate and multivariate logistic regression methodologies to identify risk factors for CPP.

**Results:**

The study included a total of 236 patients: 89 patients in the CPP group and 147 patients in the non-CPP group. No statistically significant differences were found between the two groups with regard to demographic parameters including age, height, weight, age of menarche, and number of pregnancies and births. However, the CPP group showed a higher menstrual volume score and a greater incidence of varicose veins, coupled with a lower body mass index. Transabdominal ultrasonography revealed that patients with CPP had a significantly larger diameter in the left ovarian vein (LOV) (6.2 ± 1.9 mm vs 5.0 ±2.3 mm; *P* < .05), and a higher prevalence of left internal iliac vein incompetence (21.3% vs 8.8%). Moreover, positive rates for LOV incompetence were markedly higher (94.4% vs 23.1%; *P* < .05), even in the absence of left common iliac vein compression and nutcracker phenomenon. Multivariate logistic regression analysis discerned that the LOV reflux (odds ratio [OR], 9.102; 95% confidence interval [CI], 4.578-18.099; *P* < .05), lower body mass index (OR, 0.646; 95% CI, 0.502-0.83; *P* < .05), elevated menstrual bleeding (OR, 1.182; 95% CI, 1.131-1.234; *P* < .05), and concomitant varicose veins (OR, 3.140; 95% CI, 1.067-9.273; *P* < .05) are independent risk factors for the manifestations of CPP in our patient cohorts.

**Conclusions:**

Ultrasonography serves as an efficacious modality for evaluating abdomino-pelvic vascular pathology in patients with PVVs. Notably, LOV and internal iliac vein incompetence emerge as independent risk factors for CPP, thus offering a pivotal point of reference for clinical diagnosis and therapeutic management of PVVs.

## Clinical Relevance

The identification of left ovarian vein incompetence as an independent risk factor for chronic pelvic pain (CPP) in patients with pelvic varicose veins holds significant clinical implications. This finding highlights the importance of a thorough sonographic evaluation of abdomino-pelvic veins. Recognition of the association between left ovarian vein reflux and CPP suggests that amelioration of CPP should focus on the correction of ovarian vein reflux. By prioritizing ovarian vein treatment, clinicians can potentially mitigate CPP symptoms and improve the overall quality of life for individuals with pelvic varicose veins.

**Article Highlights**

• **Type of Research**: Single-center retrospective cohort study
• **Key Findings**: Patients with chronic pelvic pain (CPP) had higher menstrual volume scores and a greater incidence of varicose veins compared with those without CPP, along with a lower body mass index. Ultrasonography showed that patients with CPP had a significantly larger diameter of the left ovarian vein (LOV) and a higher prevalence of left internal iliac vein incompetence. LOV reflux, lower body mass index, elevated menstrual bleeding, and concomitant varicose veins were identified as independent risk factors for the development of CPP.
• **Take Home Message**: Ultrasonography is a valuable tool for evaluating vascular pathology in pelvic varicose veins. LOV reflux and internal iliac vein incompetence are key independent risk factors for CPP. Addressing ovarian vein reflux may help alleviate CPP symptoms and improve patient quality of life.

Chronic pelvic pain (CPP) is defined as non-periodic pain in the lower abdomen or pelvis lasting more than 6 months. Frequently associated with the pelvic venous disorders (PVeDs), this condition presents with a complex etiology, challenging diagnosis, and sub-optimal prognosis, imposing a significant physical and psychological burden on patients.^1^ Pelvic varicose veins (PVVs) are commonly detected in gynecologic ultrasound examinations, but this sign has often been overlooked. Richet first described the correlation between CPP and PVVs in women as early as 1857.^2^ Bora et al reported that 91% of patients with PVVs had a combination of CPP, compared with 8% in the non-expanded group.^3^ Therefore, some scholars have proposed the concept of symptomatic pelvic venous insufficiency. Outflow obstruction in the inferior vena cava, renal vein, iliac vein, ovarian vein, or uterine vein may lead to PVeD, previously known as pelvic venous congestion.^4^ According to a study, the left ovarian vein (LOV) reflux emerges as a notable contributor to PVeD complicating the CPP. The utilization of a spring coil with a sclerosing agent to embolize the LOV can result in symptomatic relief rates ranging from 89% to 100%.^5^ To date, there is a lack of a large-scale systematic study on the specific risk factors for CPP in female patients with PVeD. Our study aims to retrospectively analyze 236 patients with PVV and evaluate the risk factors responsible for CPP using multifactorial regression analysis. Our overarching objective is to lay the groundwork for personalized diagnosis and treatment approaches for PVeD.

## Methods and methodology

### Patient selection

The clinical and ultrasound data were retrospectively gathered from female patients diagnosed with PVVs at our hospital between December 2017 and October 2022. Inclusion criteria were: (1) aged ≥18 years old; (2) unilateral or bilateral para-uterine and peri-vaginal vein diameters ≥4.0 mm as suggested by transvaginal ultrasound/transrectal ultrasound; and (3) voluntary completion of the Chronic Pelvic Pain Assessment Form (hereinafter referred to as the assessment form). Exclusion criteria included: (1) The patient’s symptoms, physical examination, and imaging results suggest the presence of gynecologic diseases such as endometriosis, adenomyosis, pelvic inflammatory diseases, pelvic adhesions, congenital developmental abnormalities, and tumors; (2) Patients have gastrointestinal, urologic, and spinal disorders, pubic neuralgia, and myofascial pain; (3) Previous gynecologic or pelvic surgeries; (4) History of thrombosis of the inferior vena cava and iliac veins, portal hypertension; and (5) Menstruation, pregnancy, and 1-year post-delivery. The study received approval from the Ethics Committee of Beijing Century Forum Hospital, and informed consents were obtained.

The patients were categorized into two groups: the PVV combined with the CPP group (referred to as the CPP group) and the PVV not combined with the CPP group (referred to as the non-CPP group). This grouping was determined by the responses recorded in the ‘non-periodic pelvic pain, painful intercourse, increased menstrual flow, or urgency of urination persisting for more than 6 months’ column of the Evaluation Form. Patients with responses marked as ‘Unsure’ or those with unanswered questions were excluded from the analysis.

### Clinical research methods

An assessment form was utilized, comprising the following items: (1) general data collection, including age, height, body mass index (BMI), age of menarche, number of pregnancies, number of deliveries, and assessment of menstrual flow using the menstrual blood loss chart; (2) physical examination, which records the presence or absence of varicose veins in the lower limbs, buttock, vulva, perineum, and other areas of perineum to determine the location and nature of the pain; and (3) using visual analog scoring as a criterion to quantify the severity of pain.

### Ultrasound examination and image interpretation

#### Instrumentation

Diagnostic ultrasound systems including Mindray Resona 8 and Philips Epic7c were used for imaging abdomino-pelvic veins and assessment for venous incompetence and obstruction.

Transvaginal/transrectal ultrasound procedures used an intracavitary probe frequency ranging from 4 to 8 MHz, with a fan expansion angle of 120°. During the transvaginal examination, patients were positioned semi-recumbent with the head elevated at 30°, whereas transrectal ultrasound was performed with the patient in a left-side knee-bending position. Abdomino-pelvic vessels were examined with a convex array probe with frequencies of 2.5 to 7.5 MHz, with the patient positioned at supine position, a 30° to 45° reverse Trendelenburg/upright position and left lateral decubital position depending on the veins assessed.

#### Ultrasound process

First, participants underwent transvaginal ultrasound performed by our gynecology team. This procedure assessed the size, contour, morphology, myometrial echogenicity, endothelial thickness, and double adnexal area of the uterus, as well as the myometrial and parietal blood flow, adnexal area, and pelvic floor venous plexus under color Doppler flow imaging. The internal diameter (Dv) was measured at the widest point when dilated and tortuous veins were observed.

For transabdominal examinations, they were performed by the vascular team according to our laboratory’s protocol. The renal and ovarian veins were examined in both the supine and left lateral decubitus positions, whereas the iliac veins and other pelvic varicosities were examined in the supine and 30° to 45° reverse Trendelenburg (upright) positions. The steps are shown below:

• Step 1: The left renal vein (LRV) was first identified at the space between the superior mesenteric artery and the abdominal aorta. We measured the luminal diameter of the LRV at two points: the narrowest point where the LRV crosses the gap (Da1) and the point where the renal vein dilation is most pronounced (Db1). The ratio of Db1/Da1 was calculated, with the data averaged over three measurements. Additionally, the study recorded the presence or absence of combined posterior syndrome, characterized by external compression of the LRV as it passes between the abdominal aorta and the spine.
• Step 2: We imaged the patient’s LOV longitudinally at its confluence with the LRV. We also located the LOV using the psoas major muscle as the anatomic landmark, which lies approximately 1 cm underneath. The measurement was again repeated three times with the maximum diameter recorded as Da2. We also recorded the maximum duration of regurgitation under calm breathing and during the Valsalva maneuver. Please note that during the Valsalva maneuver, the LOV draining into the LRV and treading next to the left common iliac artery (CIA) may be affected by intestinal gas, so it is recommended to measure the anterior aspect of the psoas major muscle.
• Step 3: The luminal diameter of the left common iliac vein (CIV) at the point where the right CIA crosses the left CIV was measured as Da3. The distal section of the left CIV was measured as Db3, and we calculated the stenosis ratio by Db3 − Da3/Db3.
• Step 4: Both internal iliac veins (IIVs) were imaged, and the direction of venous flow during calm breathing and the Valsalva maneuver was evaluated on color Doppler flow imaging.
• Step 5: The right ovarian vein was examined next to the inferior vena cava at the level of the umbilicus, and its diameter and direction of blood flow were measured.

Examples illustrating the measurements of the vessels are depicted in the Fig.

**Fig:**
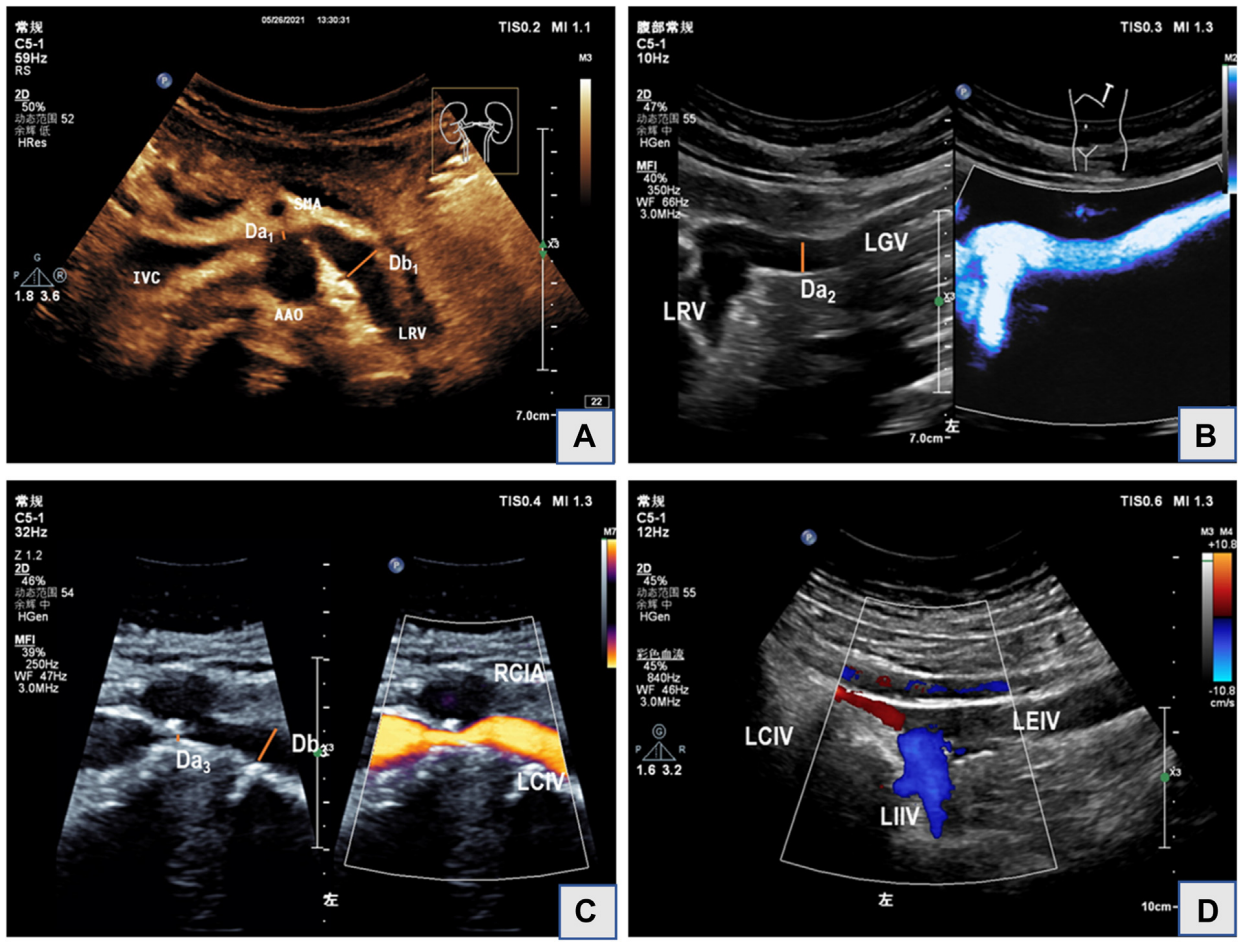
Ultrasound images of the abdomino-pelvic vessels. **A,** Nutcracker phenomenon, in which the left renal vein (*LRV*) is compressed at the space between the superior mesenteric artery (*SMA*) and abdominal aorta. (*Da1*, narrowest point at where the LRV crosses the angle between the SMA and abdominal aorta (*AAO*). *Db1*, the point at which the LRV dilation is the most pronounced.). **B,** Left ovarian vein (*LOV*) reflux. Color Doppler imaging demonstrates reversed flow within the LRV and LOV. (*Da2*, maximum diameter of LOV/left gonadal vein [*LGV*]). **C,** Left common iliac vein (*LCIV*) compression. A longitudinal view of the LCIV imaged in B-mode and color Doppler imaging shows the compression of the LCIV by the right common iliac artery (CIA) on top and vertebrae underneath. (*Da3*, the luminal diameter of the LCIV at the point where the right CIA crosses the LCIV. Db3, the distal section of the LCIV). **D,** Internal iliac vein (IIV) reflux. Color Doppler imaging shows reversed flow within the left IIV. *LEIV*, Left external iliac vein; *LIIV*, left internal iliac vein; *IVC*, inferior vena cava; *RCIA*, right common iliac artery.

#### Criteria for interpretation of abnormal ultrasound findings

(1) Nutcracker phenomenon: First, the gap between the superior mesenteric artery and abdominal aorta was significantly reduced, resulting in compression of the LRV and dilation of its distal segment. Second, after 20 minutes of spinal extension, the Db1/Da1 was more than four times higher. Additionally, blood flow rates were markedly accelerated at the site of compression; the peak velocity of the LRV at the aortomesenteric portion is approximately five times higher than that at the hilar portion.^6^ A positive diagnostic result is indicated when there is an agreement between the measurements taken in the supine and left lateral decubitus positions;
(2) Ovarian vein incompetence: Reversed flow of duration ≥0.5 seconds observed during calm breathing or the Valsalva maneuver^7^;
(3) Iliac vein compression: The left CIV is narrowed due to compression by the right CIA and vertebral body. This results in luminal stenosis of ≥50% with the opening of the iliac collateral veins in the distal segment of the compressed vein. A positive diagnostic result is indicated when there is an agreement between the supine and upright positions^8^;
(4) IIV incompetence: Reflux is detectable during calm breathing or a Valsalva maneuver^9^;
(6) PVVs: The presence of tortuous and dilated veins with a diameter (Dv) of ≥4 mm in the uterine and para-vaginal veins. Blood stagnation (<3 cm/s) with reflux induced by the Valsalva maneuver and the presence of dilated arcuate veins in the uterine myometrium communicating with PVV are also indicative of this condition.^10^

The above findings were reviewed by two sonologists both with more than 10 years of experience and discussed to reach a consensus diagnosis.

### Statistical treatment

The data were analyzed using the IBM SPSS19 software package with a two-sided test and α = .05. A statistically significant difference was considered when *P* < .05. For continuous variables, the Kolmogorov-Smirnov normality test was used first. Variables that conformed to normal distribution were expressed as mean ± standard deviation, and independent Student *t*-tests were used to compare the two groups. Variables that did not conform to a normal distribution were expressed as median (interquartile range). Comparisons between the two groups were made using the Wilcoxon rank sum test. Comparisons between groups of unordered categorical variables were analyzed using the χ^2^ test or the Fisher exact test. Independent risk factors for PVV combined with CPP were analyzed using multifactorial logistic regression for variables that were statistically significant by univariate tests.

## Results

### Characteristics of clinical data

A total of 236 female patients diagnosed with PVV were enrolled, with their age ranging between 19 and 58 years (mean age 35 ± 9.6 years). The study included 89 patients in the CPP group and 147 patients in the non-CPP group. The proportion of symptomatic PVV was 37.7%, with a visual analog score of 6.2 ± 3.1 in the CPP group. As shown in Table I, there were no statistically significant differences between the two groups in terms of age, height, weight, age of menarche, pregnancy, delivery, polycystic ovary detection rate, and combined hemorrhoids (all *P* >.05). However, the menstrual flow scores and incidences of varicose veins in the CPP group were higher than those in the non-CPP group, whereas the BMI was lower than that in the non-CPP group, with a statistically significant difference (*P* < .05).

**Table I:** Comparison of general clinical data and ultrasound data of the two groups of patients

Characteristics	CPP group (n = 89)	Non-CPP group (n = 147)	Statistic	*P* value
Age, years	31.9 ± 8.0	33.8 ± 10.1	1.50^b^	.13
Height, cm	163.3 ± 5.9	162.1 ± 5.7	1.55^b^	.12
Weight, kg	60.9 ± 9.2	61.5 ± 9.7	0.47^b^	.64
BMI, kg/m^2^	22.65 ± 1.98	23.92 ± 1.97	2.79^b^	**<.05**
Age of menarche, years	14.8 ± 1.5	14.9 ± 1.6	0.48^b^	.63
Menstrual flow, mL	76.9 (37-102)	44 (24-91)	7.93^c^	**<.05**
No. pregnancies	1.9 ± 1.4	1.8 ± 1.2	0.58^b^	.56
No. births	1.5 ± 0.3	1.4 ± 0.5	1.71^b^	.09
Polycystic ovaries	11 (12.3)	15 (10.2)	0.26^a^	.61
Combined varicose veins	27 (30.3)	22 (14.9)	7.96^a^	**<.05**
Combined hemorrhoids	40 (44.9)	61 (41.4)	0.27^a^	.60
Uterine and para-vaginal vein diameter, mm	6.9 ± 2.1	6.5 ± 3.8	0.92^b^	.36
LOV diameter, mm	6.2 ± 1.9	5.0 ± 2.3	4.14^b^	**<.05**
LOV incompetence	84 (94.4)	34 (23.1)	112.58^a^	**<.05**
Left IIV incompetence	19 (21.3)	13 (8.8)	7.40^a^	**<.05**
Left CIV compression ≥50%	31 (34.8)	41 (27.8)	1.26^a^	.26
Nutcracker phenomenon	40 (44.9)	51 (34.6)	2.46^a^	.12

*BMI,* Body mass index; *CIV,* common iliac vein; *CPP,* chronic pelvic pain; *IIV,* internal iliac vein; *LOV,* left ovarian vein.

Data are presented as number (%), mean ± standard deviation, or median (interquartile range).

Boldface *P* values indicate statistical significance.

a χ^2^ test or Fisher exact test.

b Independent sample *t*-test.

c Wilcoxon rank sum test.

### Comparison of ultrasound parameters between the two groups

The results of transabdominal ultrasound indicated that patients in the CPP group had a wider diameter of the LOV (*P* < .05), a higher positive rate of the LOV incompetence (*P* < .05), and a higher positive rate of the left IIV incompetence (*P* < .05). However, there were no statistically significant differences in the degree of left CIV compression and the phenomenon of nutcracker between the two groups (*P* > .05). Please refer to Table I.

### Multi-factor logistic regression analysis of PVV combined with CPP

The results of the multifactorial logistic regression analysis indicate that LOV reflux (odds ratio [OR], 9.102; 95% confidence interval [CI], 4.578-18.099; *P* < .05), low BMI (OR, 0.646; 95% CI, 0.502-0.83; *P* = .001), increased menstrual flow (OR, 1.182; 95% CI, 1.131-1.234; *P* < .05), and concomitant varicose veins (OR, 3.140; 95% CI, 1.067-9.273; *P* = .038) are independent risk factors for concurrent CPP in patients with PVV (Table II).

**Table II:** Logistic regression analysis of risk factors for concurrent chronic pelvic pain (CPP) in patients with pelvic varicose veins (PVVs)

Characteristics	β value	SE value	Wald value	*P* value^a^	OR value	95% CI	
						Lower limit	Upper limit
Constant	−4.22	3.24	1.70	.02			
BMI, kg/m^2^	−0.44	0.13	11.61	**.00**	0.65	0.65	0.50
Menstrual flow, mL	0.17	0.02	56.99	**<.05**	1.18	1.13	1.23
Luminal diameter of the LOV, mm	0.18	0.13	1.90	.17	1.20	0.93	1.54
Varicose veins (1 = yes; 0 = no)	1.14	0.55	4.32	**.04**	3.14	1.07	9.24
LOV incompetence (1 = yes; 0 = no)	2.21	0.35	39.66	**<.05**	9.10	4.58	18.10
Left IIV incompetence (1 = yes; 0 = no)	0.53	0.71	0.56	.46	1.70	0.42	6.85

*β,* Regression coefficient; *BMI,* body mass index; *CI,* confidence interval; *IIV,* internal iliac vein; *LOV,* left ovarian vein; *OR,* odds ratio; *SE,* standard error.

Boldface *P* values indicate statistical significance.

a Logistic regression analysis.

## Discussion

Chronic visceral pain from vascular mechanisms (CVP-vm) is defined as pain in the internal organs caused by changes in the arterial and venous blood vessels of the visceral organs in the head, neck, thorax, abdomen, and pelvic region, or by changes in the function of the vascular system or disease elsewhere in the body.^11^ In 2018, the World Health Organization updated the International Classification of Diseases (ICD-11), which now includes CVP-vm as a standalone condition in the classification catalogue. Although gynecologic disorders were previously believed to be the primary cause of CPP in women, approximately 30% of patients do not experience symptomatic improvement after treatment. In 1949, Taylor suggested that PVV was a cause of CPP. Recent findings have confirmed that approximately one-third of women with CPP have CVP-vm as the cause of the disease.^12^

The use of imaging has confirmed the close associations between PVV and CPP in women. Gultasli et al found that approximately 30% of women with unexplained CPP had PVV detected on computed tomography (CT) scans, which could explain the cause of their pain.^13^ Motta et al also detected PVV in 17 of more than 1800 women who had no pelvic or abdominal positive manifestations on CT scans. Of these, 12 suffered from CPP, resulting in an incidence of almost 70%.^14^ Bora et al found that 51 of 56 patients (91%) with PVV detected by transabdominal ultrasound had comorbid CPP, compared with only 8% in the non-dilated group.^3^ In our study, we found that 89 of 236 patients with PVV had concomitant CPP, with an incidence of 37.7%. The percentage in our study is significantly lower than the reported results in the literature. This variance could arise from the specific diagnostic positioning used in our study. The horizontal position used during CT scans might induce less vasodilatation and diminish reflux in some patients, potentially leading to false-negative results. In contrast, our study utilized positive diagnostic results by considering variations in diameter across the supine, lateral decubitus, and upright positions to minimize false-negative measurements.

In terms of patient selection, all participants in this study underwent transvaginal and/or transrectal ultrasound, with a confirmed diagnosis of PPV. The intracavitary approach provides higher resolution images, which is particularly advantageous for obese patients, as it minimizes interference from intestinal gases and reduces positional shifts during deep breathing, significantly improving diagnostic specificity.^15^ In contrast, previous studies primarily recruited patients from vein clinics or PVeD treatment centers, where most presented with PVeD or lower limb varicose veins. However, this study was conducted in a general hospital setting, where the majority of patients had no vein-related symptoms, providing a more representative sample of the broader health care landscape. Differences in prevalence rates between countries may also play a role. For instance, in nations like New Zealand and South Africa, the prevalence of PVeD can reach 26.8% among women with high fertility, whereas in countries with lower fertility rates, such as the United Kingdom, it is around 3.8%.^16^^,^^17^

Pelvic veins have unique anatomic features, and women are affected by cyclic hormonal changes and maternal factors, resulting in more intricate hemodynamics. However, the correlation between pelvic venous insufficiency and pain remains unclear; according to some research, there is a significant correlation between the diameter of the pelvic venous plexuses and the complaint of dyspareunia.^18^ Some suggest that dilatation of the ovarian vein triggers pain receptors on the vein wall; yet this hypothesis is contentious within the industry. Alternatively, it is suggested that ovarian vein reflux serves as the primary source of pain.^19^ Multifactorial logistic regression analysis in our study showed that LOV reflux, low BMI, increased menstrual flow, and concomitant varicose veins were independent risk factors for concomitant CPP in female patients with PVV. Of these, LOV reflux is the most important factor. Health care professionals, including clinicians and sonologists, should be familiar with the mentioned risk factors. They should emphasize querying patients about menstrual flow during consultation. Additionally, they should conduct thorough physical examinations, not only focusing on varicose veins in typical areas but also examining less common regions such as the buttock, vulva, perineum, and lower limbs.

Treatments of PVV complicated with CPP include medications (ie, psychotropic drugs, hormones, vasoactive drugs), surgical treatment (ie, retroperitoneal ovarian vein ligation), and interventional treatment (sclerotherapy, transcatheter embolization).^20^ Accurate diagnosis of ovarian vein reflux is essential for selecting the appropriate treatment to effectively alleviate symptoms. Transcatheter embolization of the LOV is the most effective therapeutic option for treating PVV. Combined with foam sclerotherapy, it is believed to yield better results. Embolization effectively achieves full closure of the ovarian vein and eliminates venous stasis within pelvic venous networks, subsequently relieving patient symptoms. Although detection of LOV reflux during ultrasound examinations can be challenging, the use of a semi-recumbent position can facilitate venous blood filling without the need for a Valsalva maneuver. The diagnostic accuracy of detecting reflux utilizing semi-recumbent transabdominal ultrasound can reach 90% or higher.^21^ Additionally, attention should be also paid to the characteristics of the vascular course during the sweep; renal vein confluence, anterior lumbar muscle, and the adjacent left iliac artery can be used as the starting point of the initial scan.

This study also found that the compressions of the LRV and left CIV were not independent risk factors for CPP complications in patients with PVV, which is consistent with previous research.^22^ These findings suggest that LOV and left IIV reflux play pivotal roles in CPP and should be the main targets during ultrasound examination and treatment.

Compared with other studies, our research had a larger sample size and used standardized and systematized ultrasound imaging techniques. However, the study has several limitations. Firstly, it was a cross-sectional retrospective analysis, which means that the causal relationship between PVV and CPP is unable to be determined. Secondly, the study excluded patients with vague symptoms, gynecologic diseases, and history of ilio-caval thrombosis. Furthermore, the number of patients with multiple pregnancies and births was smaller due to the influence of previous fertility policies and cultural beliefs. Thus, patient cohorts may introduce selection bias into the study. Finally, the concept of PVeD remains controversial, and there is no standardized sonographic diagnostic criterion for the condition. We addressed this challenge by assessing the abdomino-pelvic veins in different patient positions.

## Conclusions

Ultrasonography serves as an efficacious modality for evaluating abdomino-pelvic vascular abnormalities in patients with PVV. Notably, venous incompetence involving the LOV and left IIV emerges as an independent risk factor for CPP, thus offering a pivotal point of reference for clinical diagnosis and therapeutic management of PVV.

## Author Contributions

Conception and design: BZ, GL, YL

Analysis and interpretation: BZ, GL, YL

Data collection: BZ, YL

Writing the article: BZ, GL, YL

Critical revision of the article: BZ, GL, YL

Final approval of the article: BZ, GL, YL

Statistical analysis: BZ, GL, YL

Obtained funding: Not applicable

Overall responsibility: YL

## Funding

This research project received a grant from 10.13039/100015860China State Railway Group Co, Ltd. However, there was no involvement in any aspect of the research process.

## Disclosures

None.

## Appendix (online only)

*The CME exam for this article can be accessed at**http://www.jvsvenous.org/cme/home*.
